# Profil clinique et facteurs associés au syndrome de l’intestin irritable chez les étudiants en médecine à Cotonou, Bénin

**DOI:** 10.11604/pamj.2018.31.123.16336

**Published:** 2018-10-19

**Authors:** Jean Sehonou, Leoubou Roger Samuel Dodo

**Affiliations:** 1 Clinique Universitaire d’Hépato-gastroentérologie, CNHU Hubert Koutoukou Maga, Cotonou, Bénin; 2 Centre National Hospitalier et Universitaire Hubert Koutoukou Maga de Cotonou CNHU-HKM, Bénin

**Keywords:** Syndrome de l´intestin irritable, stress, anxiété, Cotonou, Bénin, Irritable bowel syndrome, stress, anxiety, Cotonou, Benin

## Abstract

**Introduction:**

Le syndrome de l'intestin irritable (SII) est un trouble fréquent qui motive souvent une consultation en médecine générale et en gastroentérologie. L'objectif de l'étude était de déterminer la prévalence du SII, décrire ses caractéristiques cliniques, déterminer ses facteurs associés et la répercussion sur la scolarité chez les étudiants en médecine de Cotonou.

**Méthodes:**

Cette étude transversale descriptive et analytique était menée au cours de la période du 1^er^ août au 29 septembre 2017, chez les étudiants en médecine. Les critères diagnostiques étaient ceux de Rome IV, l'échelle de Bristol, échelle de Cungi et HADS score. L'analyse des données était effectuée grâce au logiciel SPSS 20.0.

**Résultats:**

Sur un total de 315 étudiants inclus, 44 (14%) avaient un SII. Les facteurs associés du SII étaient le sexe féminin (OR [IC95%] = 2,4 [1,2-4,7]; p = 0,00), la prise régulière d'aliment gras (OR [IC95%] = 2,0 [1,1-3,9]; p = 0,03), un état de stress élevé à sévère (OR [IC95%] = 2,2 [1,1-4,7]; p = 0,02) et un état d'anxiété modéré à sévère (OR [IC95%] = 1,9 [0,9-3,6]; p = 0,04). L'absentéisme pour SII était rare (1 cas; 2,3%).

**Conclusion:**

Le SII est fréquent chez les étudiants en médecine de Cotonou. Les facteurs associés modifiables identifiés étaient le stress, l'anxiété et la consommation régulière des aliments gras. Aucune répercussion notable sur la scolarité n'était notée.

## Abstract (en)

**Introduction:**

irritable bowel syndrome (IBS) is a common disorder that often results in general medicine and gastroenterology consultations. This study aims to determine IBS prevalence, to describe its clinical features, to determine its associated factors as well as its impact on the education among medical students in Cotonou.

**Methods:**

We conducted a cross-sectional, descriptive and analytical study on medical students over the period 1 August-29 September 2017. The diagnostic criteria were: Rome IV, Bristol scale, Cungi scale and HADS score. Data analysis was performed using SPSS 20.0 software.

**Results:**

Out of 315 students included in the study, 44 (14%) had IBS. The factors associated with IBS were female gender (OR [CI 95%] = 2.4 [1.2 - 4.7]; p = 0.00), regular consumption of fatty foods (OR [CI 95%] = 2.0 [1.1 - 3.9]; p = 0.03), high to severe levels of stress (OR [CI 95%] = 2.2 [1.1 - 4.7]; p= 0.02) and moderate to severe state of anxiety (OR [CI 95%] = 1.9 [0.9 - 3.6]; p= 0.04). IBS-related absenteeism was rare (1 case; 2.3%).

**Conclusion:**

IBS is common among medical students in Cotonou. The identified modifiable factors associated with IBS were stress, anxiety and regular consumption of fatty foods. No appreciable impact on the education was reported.

## Introduction

Le syndrome de l'intestin irritable (SII) est un trouble fonctionnel récidivant, défini par des critères diagnostiques basés sur des symptômes et sur l'absence d'une cause organique démontrable [1, 2]. Actuellement, le diagnostic est basé sur les critères de Rome IV [3]. C'est le trouble gastro-intestinal le plus fréquent dans le monde avec une prévalence mondiale située entre 10-15%. Il est rapporté dans la littérature une prévalence plus élevée de l'affection chez les étudiants en général et particulièrement ceux en médecine [4]. Deux études menées en Algérie sur le SII, l'une en population générale [4] une prévalence à 5,8% et l'autre [5] menée chez les étudiants en médecine une prévalence de 31,2%. En effet les étudiants en médecine forment un groupe d'étudiants, caractérisés par des changements cognitifs et émotionnels énormes causés par des études et des examens de plus en plus difficiles. Cela fait d'eux des sujets à risque élevé de développer un SII. Cette affection est source de morbidité importante car elle altère souvent la qualité de vie des personnes qui en souffrent. Cette altération est corrélée à la sévérité des symptômes en particulier de la douleur causant un présentéisme ou un absentéisme voire des arrêts de travail [6, 7]. Au Bénin, aucune étude traitant le SII chez des étudiants en utilisant les critères de Rome IV n'est disponible. Elle pourrait permettre de connaitre ceux qui en souffrent afin d'organiser leur prise en charge. Les objectifs de ce travail étaient de déterminer la prévalence du SII, de décrire ses caractéristiques cliniques et de déterminer ses facteurs associés chez les étudiants en médecine à Cotonou.

## Méthodes

**Cadre, nature et période d'étude:** L'étude avait pour cadre, le Centre National Hospitalier et Universitaire Hubert Koutoukou MAGA de Cotonou (Bénin) où ces étudiants étaient en stage. Il s'agissait d'une étude transversale descriptive et analytique, réalisée chez les étudiants en faculté de médecine sur une période allant du 1^er^ aout 2017 au 29 septembre 2017.

**Population d'étude:** Etaient inclus tous les étudiants inscrits en médecine et présents au cours de la période de l'étude et en bonne santé apparente. Ces étudiants étaient ceux de la deuxième année en sixième année ayant donné leur consentement éclairé pour la participation à l'étude. Ceux ayant une pathologie organique gastro-intestinale en cours et gynécologique pouvant donner des symptômes semblables à ceux du SII n'étaient pas inclus. N'étaient inclus non plus les étudiants de la première année (il était jugé qu'ils n'étaient pas encore à même de parler avec recul du stress des études médicales).

**Echantillonnage:** La taille minimale de la population était déterminée par la formule de SCHWARZ.

$$n=\frac{\left({\text{i}}^{2}*p*q\right)}{{\text{a}}^{2}}$$

Avec i = niveau de confiance à 95% (i = 1,96); p = prévalence estimé du SII d'après une enquête antérieure [8] (p = 24,5 %); q = 1-p (q= 85%); α = marge d'erreur à 5% (α = 0,05). n = (1,962 X 0,245X 0,755)/0,052; n = 284. Nous avions ajouté 10% à l'effectif obtenu en tenant compte des erreurs de réponses et des non réponses pour avoir l'effectif final.

N = n + (n*10%); N = 284 + (284*0,1); N = 312

Pour des raisons de stratification, nous avions ramené l'échantillon à 315. Au cours du travail sur le terrain, pour l'inclusion de toutes les couches de 2^e^ année jusqu'en 6^e^ année de médecine; la taille de l'échantillon de chaque entité était déterminée par la méthode de répartition proportionnelle. Chaque entité de médecine avait un effectif **N’ = 315/5**; Soit **N’ = 63** participants.

**Collecte des données et variables étudiées:** Les données étaient recueillies sur un questionnaire standard conçu à cet effet. Les variables étudiées étaient: les données sociodémographiques, les données relatives au diagnostic du SII retenu sur la base des critères de ROME IV [3], les données cliniques, les données relatives aux perturbations de la vie universitaire et les données psychologiques comme le stress et l'anxiété. Le stress était évalué par l'échelle de stress de Cungi [9] et l'anxiété était évaluée grâce à l'échelle HADS score [4].

**Saisie et analyse statistique:** Elles étaient effectuées à l'aide du logiciel SPSS version 20.0. La comparaison des variables qualitatives était faite à l'aide du test du Chi carré. Une valeur p ≤ 0,05 était considérée comme significative.

**Considérations éthiques:** Pour garantir la confidentialité des données, les fiches ont été conçues de façon à respecter l'anonymat des étudiants. De plus, le consentement éclairé de l'étudiant était obtenu avant toute participation à l'étude.

## Résultats

**Caractéristiques générales de la population:** La population d'étude était constituée de 315 étudiants de 2^e^année jusqu'en 6^e^ année de médecine. L'âge moyen de l'ensemble des étudiants était de 21,9 ± 2 ans avec des extrêmes de 15 ans et 29 ans. Elle était constituée de 182 hommes (57,8%) et de 133 femmes (42,2%). La sex-ratio était de 1,36. Sur le plan psychologique, un état de stress élevé chez 52 cas (16,5%) était noté selon l'échelle de Cungi. Un niveau d'anxiété moyen était noté chez 36 cas; (11,4%) et sévère chez 18 cas (5,7%). Les détails des caractéristiques de la population générale sont résumés dans le Tableau 1.

**Tableau 1:** Répartition des sujets en fonction des caractéristiques générales

	Effectif (n = 315)	Pourcentage (%)
**Tranche d’âge**		
< 20 ans	38	12,1
20-25 ans	266	84,4
> 25 ans	11	3,5
**Sexe**		
Masculin	182	57,8
Féminin	133	42,2
**Perturbations psychologiques**		
**Degrés de stress**		
Très bas	81	25,7
Bas	179	56,8
Elevé	52	16,5
Très élevé	3	1
**Degré d’anxiété**		
Normal	165	52,4
Modéré	96	30,5
Moyen	36	11,4
Sévère	18	5,7

**Prévalence du SII:** Sur les 315 enquêtés, 44 étudiants avaient un SII selon les critères de Rome IV. La prévalence globale du SII était de 14%.

**Aspects épidémiologique du SII:** La population des étudiants avec un SII était constituée de 17 hommes (38,6%) et de 27 femmes (61,4%). La sex-ratio était de 0,62. L'âge moyen des étudiants diagnostiqués du SII était de 21,6 ans ± 2,2 avec des extrêmes de 15 ans et 27 ans.

**Aspects cliniques:** Au plan clinique, les symptômes digestifs présentés par ces étudiants ayant un SII étaient l'inconfort abdominal (59,1%), le ballonnement abdominal (20,4%), les borborygmes (38,6%), l'émission fréquente de gaz (56,8%). Ces étudiants présentaient des troubles du transit (de l'exonération). L'exonération était décrite comme facile (38,6) ou laborieuse (29,5% des cas). A l'aide de l'échelle de Bristol, les différents types de SII notés chez ces étudiants étaient le SII à constipation prédominante (36,4%), le SII à diarrhée prédominante (25,0%), le SII mixte (11,3%), le SII non classé (27,3%). L'émission fréquente de gaz (38,6%) et les gargouillements (29,5%) représentaient les principales gênes. Sur le plan psychologique le stress selon l'échelle de Cungi chez les étudiants ayant un SII était élevé chez 10 cas (22,7%) et très élevé dans 3 cas (6,8%). Le niveau d'anxiété moyen était noté dans 8 cas (18,2%) et sévère dans 3 cas (6,8%). Les détails des caractéristiques cliniques sont résumés dans les Tableau 2 et Tableau 2 (Suite).

**Tableau 2:** Répartition des sujets ayant un syndrome de l’intestin irritable (SII) en fonction des caractéristiques cliniques

	Effectif n= 44	Fréquence (%)
**Symptômes digestifs**		
Inconfort abdominal	26	59,1
Ballonnement abdominal	9	20,4
Borborygme	17	38,6
Emission fréquente de gaz	25	56,8
**Qualité de l’exonération**		
Facile	17	38,6
Laborieuse	13	29,5
Impérieuse	6	13,6
Satisfaisante	8	18,2
Non satisfaisante	6	13,6
**Type de SII**		
SII constipation prédominante	16	36,4
SII diarrhée prédominante	11	25,0
SII forme Mixte	5	11,3
SII non classé	12	27,3
**Comorbidité**		
Dyspepsie	5	11,4
Asthénie fréquente	19	43,2
Migraine	8	18,2
RGO	5	11,4
**Impact sur la qualité de vie**		
Arrêt de travail	0	0
Absentéisme	1	2,3
Emission fréquente de gaz	25	56,8
Gargouillement	9	20,4

**Tableau 2 (Suite):** Répartition des sujets ayant un syndrome de l’intestin irritable (SII) en fonction des caractéristiques cliniques

	Effectif (44)	Fréquence (%)
**Perturbations psychologiques**		
**Degré de stress**		
Très bas	7	15,9
Bas	24	54,6
Elevé	10	22,7
Très élevé	3	6,8
**Degré d’anxiété**		
Normal	17	38,6
Modéré	16	36,4
Moyen	8	18,2
Sévère	3	6,8

**Facteurs associés au SII:** En analyse univariée, sur le plan épidémiologique, le sexe féminin était un facteur associé au SII; les femmes avaient 2,4 fois le risque d'avoir un SII (OR [IC_95%_] = 2,4 [1,2 - 4,7]; p = 0,00). Considérant le style de vie, il n'y avait pas de relation entre l'activité physique, le temps de sommeil, la consommation d'alcool et la survenue du SII. Sur le plan des habitudes alimentaires, il n'y avait pas de relation entre la prise régulière d'une alimentation salée, d'une alimentation sucrée, de produits laitiers et la survenue d'un SII. Seuls les étudiants prenant régulièrement une alimentation grasse avaient deux fois le risque d'avoir un SII (OR [IC_95%_] = 2,0 [1,1 - 3,9]; p = 0,03). Considérant les perturbations psychologiques les sujets ayant un stress élevé à sévère (OR [IC_95%_] = 2,2 [1,1 - 4,7]; p = 0,02) et ceux ayant une anxiété modérée à sévère (OR [IC_95%_] = 1,9 [0,9 - 3,6]; p = 0,04) avaient respectivement 2,2 et 1,9 fois le risque d'avoir un SII. Les détails des facteurs associés en analyse univariée sont résumés dans les Tableau 3 et Tableau 3 (Suite). Comme le montre le Tableau 4, en analyse multivariée, seul le sexe féminin est le prédicteur indépendant le plus important de la survenue du SII.

**Tableau 3:** Répartition des sujets en fonction des facteurs associés ou non au SII en analyse univariée

	Présence du SII	Absence du SII	OR [IC_95%_]	P
	*(n = 44)*	(n = 271)		
**Sexe**				**0,00**
Féminin	27 (61,4%)	106(39,1%)	2,4 [1,2 - 4,7]	
Masculin	17(38,6%)	165(60,9%)	1	
**Tanche d’âge**				0,84
< 20 ans	6(13,6)	32(11,8)	1,8[0,2-17,4]	
20-25 ans	37(84,1)	229(84,5)	1,6[0,2-12,9]	
> 25 ans	1(2,3)	10(3,7)	1	
**Entité**				0,41
2^e^ année	7(15,9)	56(20,7)	0,8[0,3-2,5]	
3^e^ année	10(22,7)	53(19,5)	1,3[0,5-3,5	
4^e^ année	13(29,6)	50(18,5)	1,8[0,7-4,6]	
5^e^ année	6(13,6)	57(21,0)	0,72[0,2-2,2]	
6^e^ année	8(18,2)	55(20,3)	1	
**Style de vie**				
**Activité physique**				0,9
Régulièrement	15(34,1%)	90(33,2%)	1,0[0,5-2,0]	
Irrégulièrement	29(65,9%)	181(66,8%)	1	
**Temps de sommeil < 6h**				0,61
Régulièrement	18(44,1%)	100(36, 9%)	1,2 [0,6-2,2]	
Irrégulièrement	26(55,9%)	171(63, 1%)	1	
**Consommation d’alcool**				0,91
Régulièrement	4(10%)	26(9,6%)	0,9 [0,3-2,8]	
Irrégulièrement	40(90%)	245(90,4%)	1	

**Tableau 3 (Suite):** Répartition des sujets en fonction des facteurs associés ou non au SII en analyse univariée

	Presence du SII	Absence du SII	OR [IC_95%_]	P
	*(n = 44)*	(n = 271)		
**Habitudes alimentaires**				
**Alimentation salée**			-	0,48
Régulièrement	19 (43,2%)	102 (37,6%)	1,2[0,6-2,4]	
Irrégulièrement	25 (56,8%)	169 (62,4%)	1	
**Alimentation grasse**				**0,03**
Régulièrement	18 (40,1%)	69 (25,4%)	2,0 [1,1 – 3,9]	
Irrégulièrement	26(59,9%)	202 (74,6%)	**1**	
**Prise des produits laitiers**				
Régulièrement	15 (34,1%)	87 (32,1%)	1,0 [0,5- 2,1]	0,79
Irrégulièrement	29 (65,9%)	184 (67,9%)	1	
**Alimentation sucrée**				0,97
Régulièrement	23 (52,3%)	141 (52,0%)	1,0 [0,5 -1,9]	
Irrégulièrement	21 (47,7%)	130 (48%)	1	
**Consommation de fruits**				0,22
Régulièrement	10 (22,7%)	86 (31,7%)	0,6 [0,3 – 1,3]	
Irrégulièrement	34 (77,3%)	185 (68,3%	1	
**Perturbations psychologiques**				
**Stress élevé à sévère**				**0,02**
Oui	13 (29,5%)	42 (15,5%)	2,2 [1,1 - 4,7]	
Non	31 (70,5%)	229 (84,5%)	1	
**Anxiété modérée à sévère**				**0,04**
Oui	27 (61,4%)	123 (45,4%)	1,9 [0,9 – 3,6]	
Non	17 (38,6%)	148 (54,6%)	**1**	

**Tableau 4:** Répartition des sujets en fonction des facteurs associés ou non au SII en analyse mulivariée

	Présence du SII	Absence du SII	OR [IC_95%_] ajusté	P
	(*n = 44*)	*(n = 271)*		
**Sexe**				**0,02**
Féminin	27 (61,4%)	106(39,1%)	2,5 [1,1 - 5]	
Masculin	17(38,6%)	165(60,9%)	1	
**Habitudes alimentaires**				
**Alimentation grasse**				0,27
Régulièrement	18(40,1%)	69(25,4%)	1,7 [0,8 – 3,3]	
Irrégulièrement	26(59,9%)	202(74,6%)	1	
**Perturbations psychologiques**				
**Stress élevé à sévère**				0,25
Oui	13(29,5%)	42(15,5%)	1,6 [0,7 – 3,4]	
**Non**	31(70,5%)	229(84,5%)	1	
**Anxiété modérée à sévère**				0,22
Oui	27(61,4%)	123(45,4%)	1,5 [0,7 – 3,]	
Non	17(38,6%)	148(54,6%)	1	

**Répercussion sur la scolarité:** Malgré leur fréquence, ces symptômes n'entrainaient pas une répercussion sur les activités professionnelles. Rarement, elles provoquaient absentéismes et arrêt du travail.

## Discussion

A travers un questionnaire incluant les critères diagnostiques ROME IV, l'échelle de Bristol, et deux échelles d'évaluation psychique, les étudiants en médecine étaient interrogés sur le SII. Cela nous avait permis de décrire le profil clinique du SII et de déterminer les facteurs associés à l'affection chez ces derniers. Dans notre série sa prévalence était estimée à 14%. Cette prévalence est proche de celle antérieurement décrite [10] et notamment celle de Shen L *et al* [11] (15,7%), Sumeena B *et al* [12] (16,5%) et Liu L *et al* [2] (17,4%). Par contre elle était inférieure à celle rapportée par Naeem SS *et al* [13] (28,3%) et Ibrahim NK *et al* [14] (31,8%). Cette différence pourrait s'expliquer par la variabilité des méthodes d'étude, puisque les critères diagnostiques de Rome de l'affection variaient d'une étude à l'autre. Nous avons dans notre étude utilisé les critères les plus récents. Les étudiants étaient des sujets jeunes avec un âge moyen de 21,6 ans ± 2,2. Ce résultat était superposable aux données de Wells M *et al* [15] (24,1 ans) et de Liu Y *et al* [16] (23,26 ans). Une prédominance féminine de l'affection était notée. Ce constat était superposable aux données de la littérature [2, 16]. Dans notre série, la prévalence du SII plus élevée chez les étudiants des premières années de médecine était similaire aux données de Liu Y [16]. Par contre Alaqueel MK *et al* [17] rapportaient que la prévalence du SII était croissante avec l'année d'étude. Ces résultats découlent non seulement de la complexité du cursus médical mais aussi de la perception que ces étudiants avaient dudit cursus. Sur le plan clinique, en dehors de la douleur abdominale qui était le symptôme principal, plusieurs autres troubles intestinaux étaient notés. Il s'agissait d'un inconfort abdominal (59,1%), d'une émission fréquente de gaz (56,8%), de borborygmes (38,6%) et d'un ballonnement abdominal (20,4%). Ces symptômes étaient également rapportés par certains auteurs [5, 18]. Ces symptômes étaient suivis des troubles de l'exonération. L'exonération était décrite comme facile (38,6%) ou laborieuse (29,5%) dans notre série. Ce constat est similaire à ce qui est rapporté dans de la littérature [5]. Dans notre série, la forme à constipation prédominante était la principale forme comme l'ont rapporté Dong YY *et al* [19] d'une part, Guillaume PC *et al* [10] d'autre part. Cela contrastait avec la prédominance de la forme mixte décrite par d'autres auteurs [12, 17, 18]. Les facteurs favorisants étaient les changements d'habitudes alimentaires, le grignotage et peut être les conditions d'exonérations et le stress.

Dans notre série, les perturbations psychologiques telles qu'un état de stress élevé à sévère et un état d'anxiété modéré à sévère en analyse univariée étaient des facteurs de risque de survenue du SII. Ces résultats concordaient avec les données de plusieurs auteurs [13, 14, 20-22]. Ces notions contribuent à expliquer la prédominance du SII dans les premières années de scolarité médicale. Les cours y sont denses, les emplois du temps rigoureux et l'investissement émotionnel important: c'est le début du stage pratique et des premiers contacts avec les patients à l'hôpital. Cette forte participation psychique au cours de la maladie explique pourquoi la prise en charge médicamenteuse à elle seule est difficile et souvent source d'insatisfaction pour les patients; cela pourrait justifier un recours de plus en plus fréquent à des traitements alternatifs ou complémentaires dont la médecine traditionnelle africaine [23]. Le recours aux méthodes de traitement par la pharmacopée traditionnelle africaine n'a pu être évalué dans cette étude. Dans ce milieu il est socialement dévalorisant de déclarer avoir recours aux pratiques ne relevant pas de la médecine moderne de type occidental même si ledit recours est fréquent [24]. Dans notre série le sexe féminin était également un facteur de risque du SII. Ce constat est similaire à celui d'Ibrahim NK *et al* [14]. Une hypothèse selon laquelle les hormones sexuelles féminines pourraient faciliter la survenue de l'affection a été évoquée pour expliquer ce constat [16, 25]. En outre, les femmes sont plus susceptibles d'avoir plus des problèmes psychologiques, ce qui peut être lié à la forte prévalence du SII [16]. En termes d'habitudes alimentaires, seule la consommation régulière d'aliments gras était également un facteur de risque de survenue du SII. Ce constat était également rapporté par Sang-Wook S [26]. En analyse multivariée, seul le sexe féminin était le prédicteur indépendant le plus important de la survenue du SII. Malgré leur fréquence, ces symptômes n'entrainaient pas une répercussion sur les activités professionnelles. Rarement elles provoquaient absentéismes et arrêt du travail.

## Conclusion

Le syndrome de l'intestin irritable est une affection fréquente chez les étudiants en médecine de Cotonou. La forme la plus fréquente est celle avec constipation prédominante. Les facteurs de risque modifiables identifiés sont le stress, l'anxiété et la consommation régulière d'une alimentation grasse. Une meilleure gestion du stress, l'anxiété et une réduction de la consommation des aliments gras pourraient améliorer la qualité de vie de ces étudiants. Cependant la répercussion de ce syndrome sur la scolarité était faible.

### Etat des connaissances actuelles sur le sujet

- Le syndrome de l'intestin irritable est fréquent dans la population générale y compris chez les étudiants en médecine;
- Cependant la fréquence varie d'un continent à l'autre et d'un pays à l'autre;
- Les facteurs associés sont variables.

### Contribution de notre étude à la connaissance

- C'est une des rares études menées chez les étudiants en Afrique Subsaharienne;
- Les facteurs étiologiques tels que le sexe féminin, le rôle du stress et de l'anxiété et les facteurs alimentaires ont été mis en évidence.

## Conflits d’intérêts

Les auteurs ne déclarent aucun conflit d'intérêts.
